# The mechanism of charge density wave in Pt-based layered superconductors: SrPt_2_As_2_ and LaPt_2_Si_2_

**DOI:** 10.1038/srep15052

**Published:** 2015-10-09

**Authors:** Sooran Kim, Kyoo Kim, B. I. Min

**Affiliations:** 1 Department of Physics, Pohang University of Science and Technology, Pohang, 790-784, Korea; 2 c_CCMR, Pohang University of Science and Technology, Pohang 790-784, Korea

## Abstract

The intriguing coexistence of the charge density wave (CDW) and superconductivity in SrPt_2_As_2_ and LaPt_2_Si_2_ has been investigated based on the *ab initio* density functional theory band structure and phonon calculations. We have found that the CDW instabilities for both cases arise from the q-dependent electron-phonon coupling with quasi-nesting feature of the Fermi surface. The band structure obtained by the band-unfolding technique reveals the sizable q-dependent electron-phonon coupling responsible for the CDW instability. The local split distortions of Pt atoms in the [As-Pt-As] layers play an essential role in driving the five-fold supercell CDW instability as well as the phonon softening instability in SrPt_2_As_2_. By contrast, the CDW and phonon softening instabilities in LaPt_2_Si_2_ occur without split distortions of Pt atoms. The phonon calculations suggest that the CDW and the superconductivity coexist in [*X*-Pt-*X*] layers (*X* = As or Si) for both cases.

Low-dimensional systems often suffer from intrinsic instabilities, revealing diverse interesting phase transitions upon cooling, such as charge density wave (CDW), spin density wave, superconductivity (SC), and so on. In general, those phases are detrimental to each other. Therefore the coexistence of those phases in a system has been a long-standing subject of importance in the physics of low-dimensional systems^1^,^2^,^3^,^4^,^5^,^6^. The Pt-based layered superconductors, SrPt_2_As_2_ and LaPt_2_Si_2_, of the present study belong to such quasi two-dimensional (2D) systems, which exhibit the coexistence of the CDW and the SC at low temperature (*T*)^7^,^8^. In fact, the Pt-based layered systems draw recent attention because of their structural similarity to Fe-based *A*Fe_2_As_2_ (122) (*A* = Ba, Ca, Sr, or Eu) superconductors, which have been intensively studied these days^9^,^10^,^11^,^12^,^13^,^14^,^15^,^16^,^17^.

SrPt_2_As_2_ was reported recently to be a BCS-like superconductor having two s-wave superconducting gap feature as in MgB_2_^18^. SrPt_2_As_2_ undergoes a CDW transition at T_*CDW*_ ≃ 470 K, which is accompanied by the superconducting transition at T_*c*_ ≃ 5 K. Below T_*c*_, the SC coexists with the CDW phase^7^,^19^. At high *T*, SrPt_2_As_2_ crystallizes in the tetragonal structure of CaBe_2_Ge_2_-type (*P*4/*nmm*), which is quite similar to ThCr_2_Si_2_-type structure of *A*Fe_2_As_2_ superconductors^7^. Differently from *A*Fe_2_As_2_, however, Pt and As in SrPt_2_As_2_ have reversed positions for every other layer, as shown in Fig. 1a. Namely, there are alternating [As2-Pt1-As2] and [Pt2-As1-Pt2] layers along the *c*-direction. The CDW modulation vector of SrPt_2_As_2_ was reported experimentally to be *q*_*CDW*_ = 0.62 a^*^ = (0.62, 0, 0), which yields the supercell structure with the modulation in the [As2-Pt1-As2] layers below T_*CDW*_ (see Fig. 4)^19^,^20^,^21^. Here a^*^ is the reciprocal lattice vector, a^*^ = (1, 0, 0)2*π*/*a*. Even below T_*CDW*_, SrPt_2_As_2_ has unique feature containing the split-off positions of Pt1 and As2, as shown in Fig. 1b. Interestingly, another CDW was recently observed at 255 K in [Pt2-As1-Pt2] layer too^22^.

There have been only a few band structure calculations for SrPt_2_As_2_^23^,^24^. Nekrasov *et al*.^23^ obtained the density of states (DOS) and Fermi surface (FS) of the high *T* phase of SrPt_2_As_2_ having the tetragonal structure above T_*CDW*_. They found that 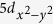 state of Pt1 is dominant at the Fermi level (E_*F*_) and the FSs are mostly 3D-like except one cylinder-like FS. Shein *et al*.^24^ investigated the energetics of three types of 122 system: CaBe_2_Ge_2_-type and two hypothetical ThCr_2_Si_2_-type structures. They reported that CaBe_2_Ge_2_-type is more stable than ThCr_2_Si_2_-type polymorphs. However, none of these studies explored the electronic structures of the low *T* phase of SrPt_2_As_2_ having the split-off positions of Pt and the CDW modulated structure.

Another Pt-based layered system LaPt_2_Si_2_ has similar physical properties to SrPt_2_As_2_. At high *T*, LaPt_2_Si_2_ crystallizes in the tetragonal structure of CaBe_2_Ge_2_-type, which is similar to Fig. 1a (Sr and As are replaced by La and Si). Upon cooling, it undergoes the CDW transition at 112 K with the CDW vector of *q*_*CDW*_ = (*n*/3, 0, 0) (*n* = 1 or 2)^8^. Nagano *et al*.^8^ suggested a CDW-induced supercell at low *T*, which corresponds to the tripling of the original unit cell. Below *T* ~ 2 K, the SC emerges in coexistence with the CDW state^8^. It was also reported that LaPt_2_Si_2_ is more stable in the CaBe_2_Ge_2_-type structure than in the ThCr_2_Si_2_-type structure^25^. The FSs of CaBe_2_Ge_2_-type structure are mostly 2D-like, while the FSs of ThCr_2_Si_2_-type structure are 3D-like. However, the unique feature of the split-off positions of Pt1 and As2 in SrPt_2_As_2_ has not been observed in LaPt_2_Si_2_.

Despite existing studies on SrPt_2_As_2_ and LaPt_2_Si_2_, there are important remaining issues. There has been no theoretical explanation on the mechanisms of the observed CDW instabilities in SrPt_2_As_2_ and LaPt_2_Si_2_. Kudo *et al*.^7^ once stated that the CDW transition of SrPt_2_As_2_ seemed to originate from the FS nesting, but they did not specify which band is responsible for the FS nesting. Above all, it has not been clarified whether the CDW instabilities in SrPt_2_As_2_ and LaPt_2_Si_2_ have the same mechanisms or not. Also, there have been no phonon studies on SrPt_2_As_2_ and LaPt_2_Si_2_, which can provide direct clue to the CDW structural transitions. On the basis of phonon studies, one can also investigate SC properties in these CDW systems.

In this letter, to address the above questions, we have investigated the CDW and SC properties of SrPt_2_As_2_ and LaPt_2_Si_2_, using the first-principles density-functional theory (DFT) band structure and phonon calculations. In SrPt_2_As_2_, the split distortions of Pt1 in [As2-Pt1-As2] layers are found to play an essential role in driving the CDW instability. This feature in SrPt_2_As_2_ is distinct from that in LaPt_2_Si_2_ that does not need the split distortions to drive the CDW instability. However, the **q**-dependent electron-phonon interaction with quasi-nesting feature of the FS is expected to drive the CDW transitions in both cases. The phonon studies also revealed that the SC emerges mainly in the CDW layer of Pt1 for both SrPt_2_As_2_ and LaPt_2_Si_2_.

## Results

**Figure 1:**
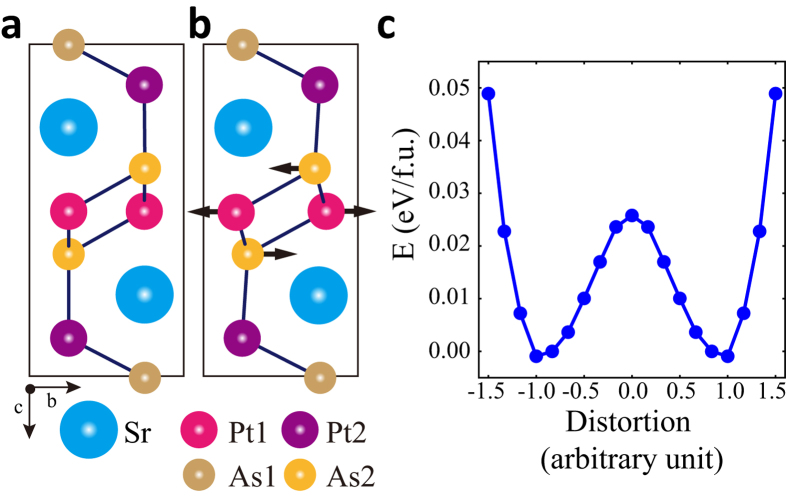
(**a**) Orthorhombic structure of SrPt_2_As_2_ with Pt1 and As2 at the ideal positions without the split distortion (no-split-SPA). (**b**) Orthorhombic structure of SrPt_2_As_2_ with Pt1 and As2 at the split-off positions (split-SPA). Black arrows in (**b**) represent the split distortions of Pt1 and As2. (**c**) Total energy variation of SrPt_2_As_2_ with respect to the split distortion (distortion = 0.0 for no-split-SPA and distortion = 1.0 for split-SPA).

To examine the mechanism of CDW instability in SrPt_2_As_2_, we focused on the role of split distortions of Pt, and so considered two structures. The first one is the orthorhombic structure in Fig. 1a without the split distortions of Pt1 and As2 (we call it “no-split-SPA” hereafter). The no-split-SPA structure is close to the tetragonal CaBe_2_Ge_2_-type structure above T_*CDW*_. The second one is another orthorhombic structure in Fig. 1b, which has the split-off positions of Pt1 and As2 (hereafter “split-SPA”). Split-SPA has the structure that is close to that of SrPt_2_As_2_ below T_*CDW*_, but does not contain the modulation by **q**_*CDW*_ = 0.62a^*^. The split-SPA structure is obtained by making the antiferro-like distortions of Pt1 and As2 and then performing the atomic relaxation. The initial structure data for SrPt_2_As_2_ before the structural relaxation was adopted from Imre *et al*.^20^. The initial lattice constant and atomic positions of LaPt_2_Si_2_ were adopted from Shelton *et al*.^26^, and Nekrasov *et al*.^23^, respectively. The relaxed structural parameters of SrPt_2_As_2_ and LaPt_2_Si_2_ are summarized in the supplement^27^.

First, we checked the energetics of SrPt_2_As_2_ with respect to the split distortion. The total energy variation from no-split-SPA to split-SPA is shown in Fig. 1c. The negative distortion means the opposite split directions of Pt1 and As2. We obtained the double-well shaped energy profile, which indicates that the split distortions of Pt1 and As2 indeed lower the total energy. The energy difference between no-split-SPA and split-SPA is Δ*E* ≃ 27 meV/f.u.

**Figure 2:**
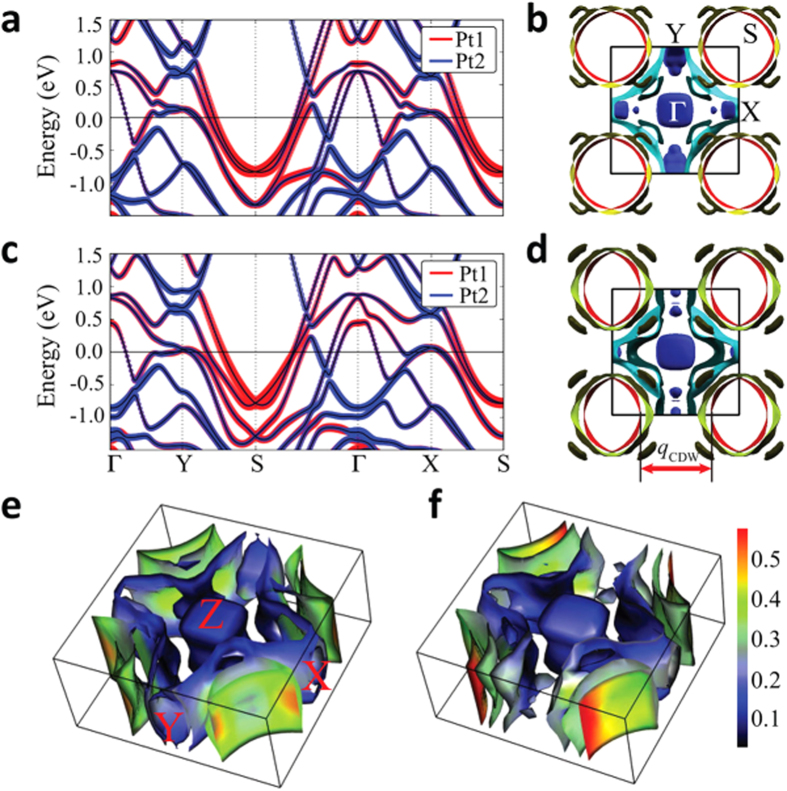
(**a**) Band structure of no-split-SPA. Pt1 and Pt2 band characters are shown with fat bands. (**b**) FS of no-split-SPA in the a^*^b^*^ plane. (**c**) Band structure of split-SPA. (**d**) FS of split-SPA in the a^*^b^*^ plane. The red FSs centered at S become flatter, which provides the nesting vector q_*CDW*_. (**e**) FS of no-split-SPA in the full Brillouin zone (BZ). (**f**) FS of split-SPA in the full BZ. Color bar for (**e**,**f**) represents the Pt1 contribution to the FS (maximum value = 1). For split-SPA, the flat-red region is seen to be enhanced at the S-centered FSs.

Figure 2 shows band structures and FSs of no-split-SPA and split-SPA. As shown in Fig. 2a,c, the main character of the dispersive bands around S is attributed to Pt1 band in both no-split-SPA and split-SPA. But the significant difference between the no-split-SPA and split-SPA is revealed in the FSs. Figure 2b,e for no-split-SPA show mainly the 3D-like FSs except one cylinder-like FS centered at S (red-colored FS in Fig. 2b), as is consistent with existing calculations^23^,^24^. Interestingly, the circular-cylindrical FS for no-split-SPA is changed into the ellipsoidal-cylindrical FS for split-SPA, as shown in Fig. 2d, and so the parallel portion of the FS is increased. Pt1-projected FSs of no-split-SPA and split-SPA are presented in Fig. 2e,f, respectively. For no-split-SPA, the FS has almost 4-fold rotational symmetry, and the Pt1 projection is distributed rather uniformly over the cylindrical FS. On the other hand, for split-SPA, the 4-fold rotational symmetry is completely broken because of the ellipsoidal-cylindrical FSs at S. It is worth noting in Fig. 2d that the nesting vector connecting the flat parts of ellipsoidal FSs is in good agreement with the experimental CDW modulation vector of *q*_*CDW*_ = (0.62, 0, 0) suggested by Imre *et al*.^20^. This result demonstrates that the split distortions of Pt1 and As2 in [As2-Pt1-As2] layer of split-SPA are essential to drive the CDW transition. Also notable feature in Fig. 2f is that the Pt1 character is dominant at longer parts of the ellipsoidal FSs, which clearly indicates that the Pt1 band is responsible for the CDW instability in split-SPA.

**Figure 3:**
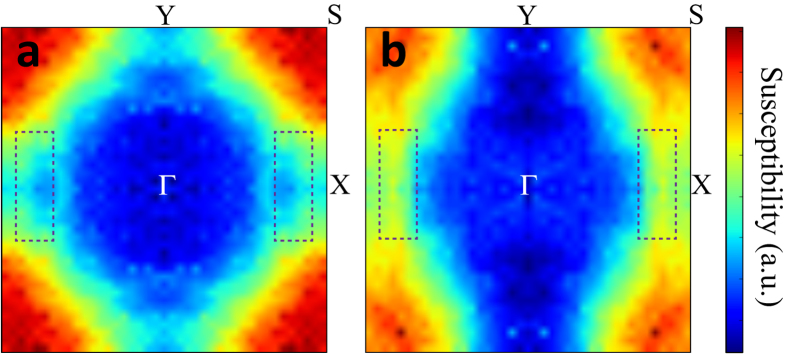
The projected charge susceptibility calculated with Pt1 matrix element for (a) no-split-SPA and (b) split-SPA. Dotted boxes represent the regimes of the observed CDW modulation vector *q*_*CDW*_ of SPA.

To check *q*_*CDW*_, we have also calculated the charge susceptibility. The real part of charge susceptibility, however, does not show the pronounced peaks, which correspond to the observed CDW vector (Data are not shown). In fact, the discrepancy between the susceptibility peak and the experimental CDW vector has been previously pointed out^28^, which shows the limitation of bare susceptibility with a constant matrix element to predict a proper CDW vector. So, we have instead calculated the projected susceptibilities in Eq. 1 having the Pt1 matrix element, which is expected to give dominating contribution to the CDW transition:


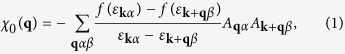


where *A*_**k***α*_ is the weight of a specific atom in the 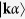 wave function. This calculation method considers the special role of a specific atom in the present case, Pt1 atom^28^. Figure 3 shows the projected susceptibilities calculated with matrix element of Pt1 for (a) no-split-SPA and (b) split-SPA. It is seen that, only for the split-SPA, the susceptibility peak appears near the experimental *q*_*CDW*_ (denoted by the dotted box), which indicates the quasi-nesting feature^28^. However, there exist other *q*-vectors having more pronounced peaks. We believe that this is the limitation of the susceptibility calculation because the susceptibility cannot capture the electron-phonon interaction, which plays and important role in the CDW transition^29^,^30^,^31^.

**Figure 4:**
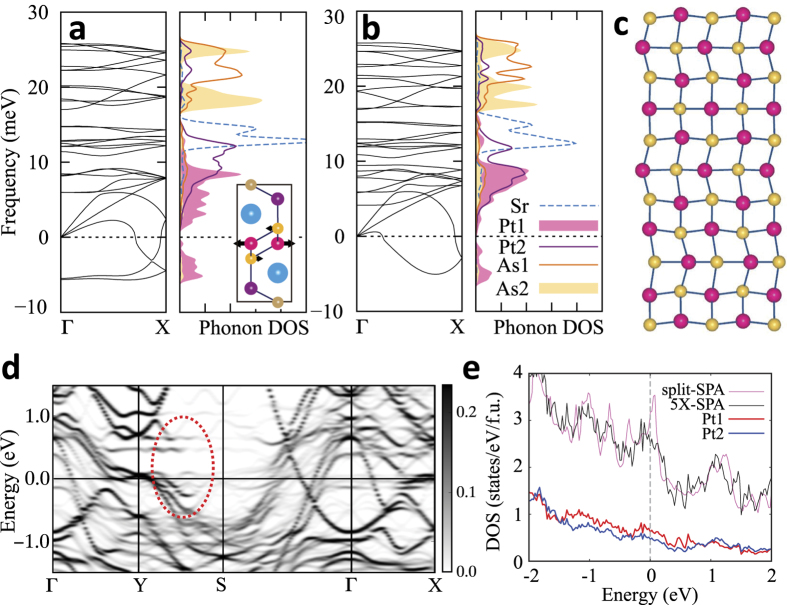
(**a**) Phonon dispersion and partial phonon DOS for no-split-SPA. (**b**) The same for split-SPA. The negative frequency here implies the imaginary phonon frequency, indicating the structural instability. In the inset of (**a**), the softened phonon mode at Γ is depicted. (**c**) The modulated structure (5X-SPA) of [As2-Pt1-As2] layer in the *ab* plane due to the softened phonon mode at **q** = (0.6, 0, 0) of (**b**). (**d**) The band structure of 5X-SPA unfolded into the BZ of split-SPA. In comparison to band structures in Fig. 2c, the partial band gap opens along YS after the CDW transition (red-circled region). (**e**) Total electron DOSs are compared between the split-SPA and 5X-SPA cases. Partial electron DOSs of Pt1 and Pt2 of 5X-SPA are also plotted.

In order to consider the electron-phonon interaction in the CDW transition, we performed phonon dispersion calculations for both no-split-SPA and split-SPA. As shown in Fig. 4a,b, the phonon softening instabilities occur in both cases, indicating the structural instabilities. This feature is consistent with experiment in that the ground state of SrPt_2_As_2_ has the CDW modulated structure^20^. The softened phonon modes arise mainly from Pt1, as shown in the partial phonon DOSs, suggesting that the CDW transition occurs in the Pt1 layers. It also suggests that the Pt1 layer has the large electron-phonon interaction. Inset of Fig. 4a shows the normal mode of softened phonon at Γ for no-split-SPA, which induces the split distortions of Pt1 and As2. This phonon mode induces the structural transition from no-split-SPA to split-SPA, which is consistent with structural energetics in Fig. 1c.

Figure 4b shows that the Γ point softening disappears for split-SPA, and the phonon softening instability becomes dominant near (0.4, 0, 0), which is equivalent to (0.6, 0, 0). This value is consistent with the experimental *q*_*CDW*_ = (0.62, 0, 0)^20^. Indeed, the relaxed structure modulated by the softened phonon mode at **q** = (0.6, 0, 0) in Fig. 4c is very close to the experimentally suggested structure after the CDW transition^20^. We will refer to this relaxed structure as 5X-SPA, as it is five-fold supercell structure due to **q** = (0.6, 0, 0). The total energy of 5X-SPA is lower than that of split-SPA by ~19 meV/f.u. Figure 4d shows the unfolded band structure of 5X-SPA into the Brillouin zone (BZ) of the split-SPA. In comparison to Fig. 2c, the partial band gap opening appears along Y-S, as indicted by red circle. This partial gap emerges due to the structural displacements modulated by the phonon mode at (0.6, 0, 0). The significant change of the electronic structure by the phonon mode reveals the sizable electron-phonon interaction of that phonon mode. The partial gap opening along Y-S is indeed consistent with the band structure calculation in Fig. 2, confirming that the Pt1 band along Y-S is responsible for the CDW instability. Therefore, the combined study of electronic structure and phonon calculations demonstrates that the CDW of SrPt_2_As_2_ originates from the **q**-dependent electron-phonon coupling with quasi-nesting feature of the FS.

Figure 4e provides the total DOSs of split-SPA and 5X-SPA, which shows that 5X-SPA is still metallic, reflecting that the CDW nesting is imperfect. Only the partial gap opens with the modulation vector, as shown in Fig. 4d. This is one reason why the CDW and the SC could coexist in SrPt_2_As_2_^1^. The DOS at E_*F*_ (N(E_*F*_)) is lower for 5X-SPA (2.46 states/eV/f.u.) than for split-SPA (3.18 states/eV/f.u.). The calculated specific heat coefficient *γ*_*cal*_ for 5X-SPA is 5.80 mJ/molK^2^. Using the experimental specific heat coefficient *γ*_*exp*_ = 9.72 mJ/molK^2^^7^, the electron-phonon coupling constant *λ* of 0.68 is obtained from *γ*_*exp*_ = *γ*_*cal*_(1 + *λ*). This moderate coupling suggests that SrPt_2_As_2_ would be a conventional BCS superconductor mediated by phonon^24^. Notable feature is that, even after the CDW transition, the contribution to the DOS at E_*F*_ comes more from Pt1 band. The ratio of Pt1 and Pt2 DOSs at E_*F*_ is ~1.3. This value would be enhanced with the consideration of the additional CDW at 255 K, which occurs in [Pt2-As1-Pt2] layer^22^. This indicates that the [As2-Pt1-As2] layer is more susceptible to the emergence of the SC.

**Figure 5:**
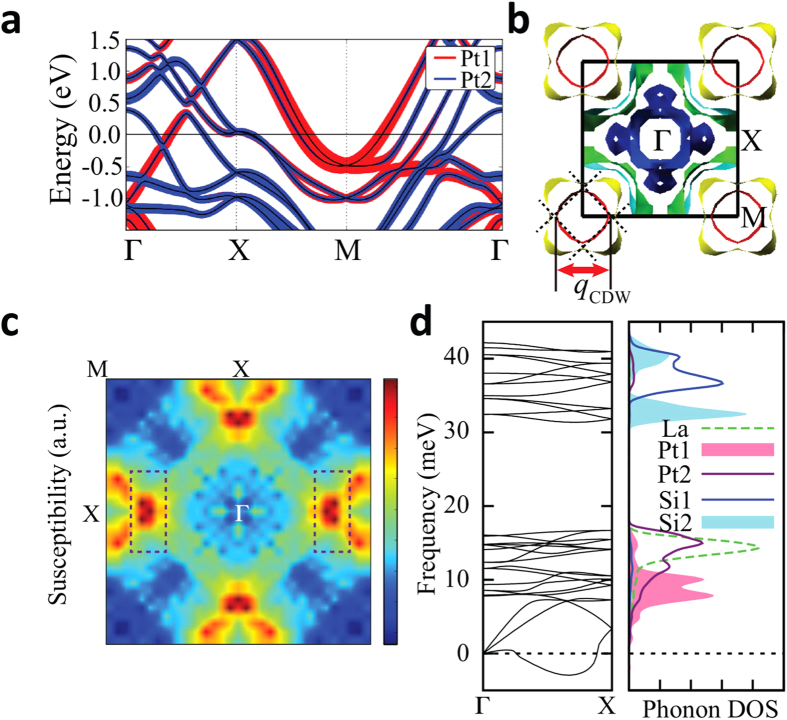
(**a**) Band structure of tetragonal LaPt_2_Si_2_ (t-LPS). Pt1 and Pt2 band characters are shown with fat band. (**b**) FS of t-LPS. The nesting vector is close to the observed q_*CDW*_. (**c**) The projected charge susceptibility with Pt1 matrix element for t-LPS. Dotted boxes represent the regimes of the observed CDW modulation vector *q*_*CDW*_ of LPS. (**d**) Phonon dispersion and partial phonon DOS of t-LPS.

For comparison, we performed the band structure and phonon calculations for tetragonal LaPt_2_Si_2_ (t-LPS). Figure 5a shows the band structure of t-LPS. The Pt1 band produces the electron pocket FSs around M. The FS in red around M, which originates from Pt1 band, quite resembles two parallel FSs, as plotted by dotted lines in Fig. 5b. In this case, the FS nesting occurs through the vector connecting the corners, which perfectly nests both parallel FSs^32^. The nesting vector indicated in Fig. 5b is quite close to the observed *q*_*CDW*_ = (1/3, 0, 0)^8^. We have also calculated the charge susceptibility. Here too, the susceptibility with constant matrix elements does not show the peak at the experimental CDW vector (Data is not shown). By contrast, the projected susceptibility with Pt1 matrix element produces the peak near *q*_*CDW*_ = (1/3, 0, 0), as denoted by the dotted box in Fig. 5c. The discrepancy between the total and projected susceptibilities implies again the quasi-nesting feature^28^.

To check the role of the electron-phonon coupling to structural transition, we have calculated the phonon dispersion curve of t-LPS too. The phonon dispersion of t-LPS in Fig. 5d contains the softened phonon mode, which is consistent with the CDW structural transition. The phonon softening occurs mainly from the Pt1, as in SrPt_2_As_2_, suggesting that the Pt1 layer has the large electron-phonon interaction. Therefore, the CDW instability in LaPt_2_Si_2_ also arises from the **q**-dependent electron-phonon coupling with quasi-nesting feature of the FS, as in SrPt_2_As_2_. But, in contrast to the case in SrPt_2_As_2_, the phonon softening at Γ causing the split distortions of Pt1 does not occur. It is compatible with the nonexistence of the split distortion in LaPt_2_Si_2_.

**Figure 6:**
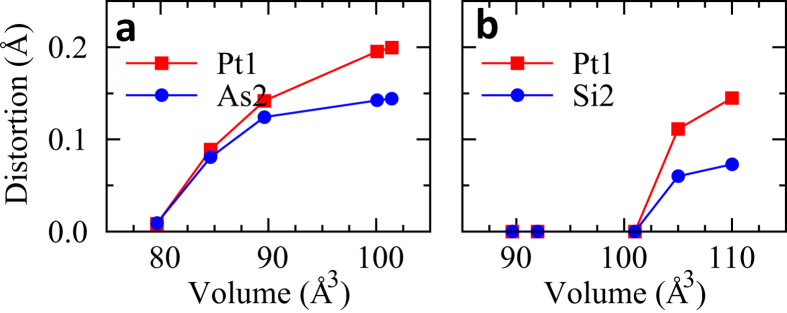
The variation of split distortion with the volume. (**a**) SrPt_2_As_2_ (**b**) LaPt_2_Si_2_. The experimental volumes are 100.08 Å^3^/f.u. for SrPt_2_As_2_ and 89.62 Å^3^/f.u. for LaPt_2_Si_2_.

The difference between LaPt_2_Si_2_ and SrPt_2_As_2_ in the split distortions is expected to arise from the volume difference between SrPt_2_As_2_ (100.08 Å^3^/f.u.) and LaPt_2_Si_2_ (89.62 Å^3^/f.u.). To investigate the volume effect, the optimized atomic positions at each volume is determined by performing the structural relaxation calculations. Figure 6 shows the split distortion sizes of Pt1 and As2 (or Si2) as a function of volume. We found that the split distortions in SrPt_2_As_2_ disappear with decreasing the volume as in Fig. 6a. In contrast, the split distortions appear in LaPt_2_Si_2_ with increasing the volume as shown in Fig. 6b. These features suggest that stability of split distortions is strongly dependent on the volume of the system. Namely, the split distortions become stable with increasing the volume in both SrPt_2_As_2_ and LaPt_2_Si_2_. We note another Pt-based superconductor, BaPt_2_Sb_2_, which also contains the deformed square lattice with the split-off Pt positions in [Sb-Pt-Sb] layer^33^. The volume of BaPt_2_Sb_2_ is 118.47 Å^3^/f.u., which is larger than those of SrPt_2_As_2_ and LaPt_2_Si_2_.

**Figure 7:**
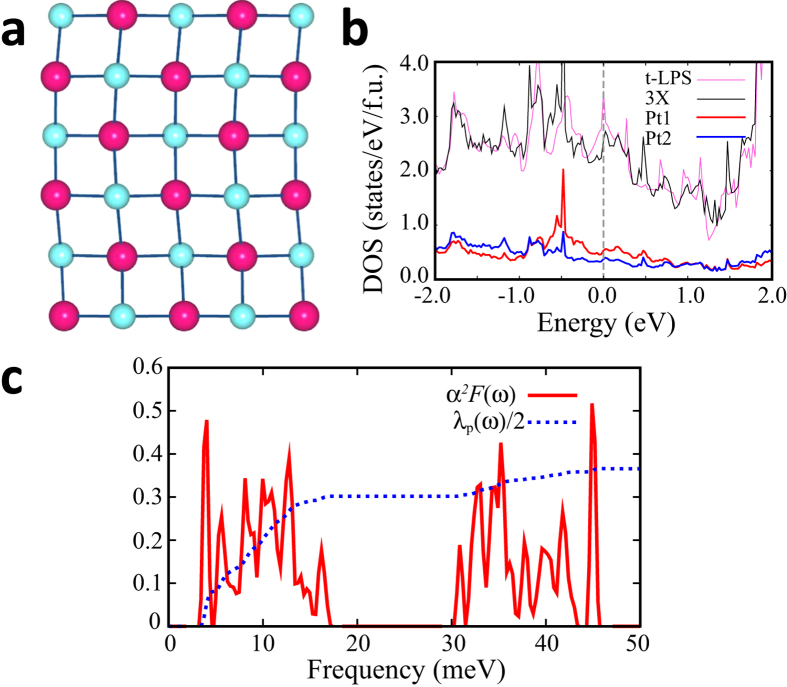
(**a**) The modified structure (3X-LPS) of [Si2-Pt1-Si2] layer in the *ab* plane due to the softened phonon mode at **q** = (1/3, 0, 0). (**b**) Total electron DOSs are compared between t-LPS and 3X-LPS. Partial electron DOSs of Pt1 and Pt2 of 3X-LPS are also plotted. (**c**) The Eliashberg function and *λ*_*p*_(*ω*) of 3X-LPS.

The phonon softening instability at *q* = (1/3, 0, 0) is consistent with the observed CDW vector *q*_*CDW*_ = (1/3, 0, 0), which produces the three-fold supercell structure. Figure 7(a) shows the relaxed structure generated by the softened phonon mode at *q*_*CDW*_ = (1/3, 0, 0). We will refer to this structure as 3X-LPS. The total energy of 3X-LPS is lower than that of t-LPS by ~2 meV/f.u. The modulations occur mainly in Pt1 layer of [Si2-Pt1-Si2], which suggests the Pt1 layer as the CDW layer, as in SrPt_2_As_2_. This result is contrary to the speculation of Nagano *et al*.^8^, who claimed that [Pt2-Si1-Pt2] layer would be the CDW layer.

Figure 7(b) presents the DOSs of t-LPS and 3X-LPS. It is seen that 3X-LPS is still metallic even with the CDW distortion. The DOS at E_*F*_ is lower for 3X-LPS than for t-LPS, which is consistent with the stabilized 3X-LPS and also with the paramagnetic susceptibility measurement^8^. The ratio of Pt1 and Pt2 DOS at E_*F*_ for 3X-LPS is ~1.4. The higher DOS at E_*F*_ for Pt1 suggests that Pt1 layer is more susceptible to the SC transition.

To identify the main superconducting layer, we performed the calculation of e-ph coupling constant, *λ*_*p*_, for 3X-LPS^27^. Figure 7(c) shows the Eliashberg function, *α*^2^*F*(*ω*), and the electron-phonon coupling constant, *λ*_*p*_(*ω*), of 3X-LPS. The peak of *α*^2^*F*(*ω*) and abrupt change of *λ*_*p*_(*ω*) appear at around ~3.9 meV. Indeed this phonon frequency yields the largest contribution to *λ*_*qν*_ at *q* = Γ. The normal mode at this frequency is mainly composed of displacements of Pt1 in [Si2-Pt1-Si2] layer, which suggests that the main contribution to the SC comes from Pt1 layers. It is noteworthy that the Pt1 layer is the CDW-modulated layer, which implies that the SC and the CDW coexist in the same layer. The arrangement of Pt in [Si2-Pt1-Si2] layer is more 2D-like than in [Pt2-Si1-Pt2] layer. It is thus expected that the 2D nature and the CDW modulation of [Si2-Pt1-Si2] layer facilitate the emergence of the SC more effectively.

Finally, we have evaluated the superconducting parameters for 3X-LPS after the CDW transition, using the Eliashberg e-ph coupling theory and the Allen-Dynes formula for the critical temperature *T*_*c*_^34^,^35^,


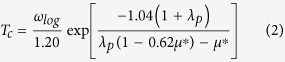


where 
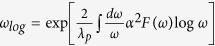
, *α*^2^*F*(*ω*) is the Eliashberg function, and μ^*^ is the effective Coulomb repulsion parameter. As provided in Table 1, we have obtained T_*c*_ = 3.5 K for μ^*^ = 0.13 (μ^*^: the effective Coulomb repulsion parameter), which is in good agreement with the observed *T*_*c*_ of ~2 K^8^,^26^. Interestingly, the calculated electron-phonon coupling constant, *λ*_*p*_, of 3X-LPS is comparable to experimentally estimated *λ* = 0.68 of 5X-SPA, whose T_*c*_ ≃ 5 K is close to *T*_*c*_ of LaPt_2_Si_2_.

## Conclusion

We have found that the CDW instabilities in both SrPt_2_As_2_ and LaPt_2_Si_2_ arise from the electron-phonon couplings with the quasi-nesting feature of the FSs. But the former takes place in the presence of the split distortions of Pt1 atoms, while the latter in the absence of the split distortions. These features are corroborated by the projected charge susceptibilities with Pt1 matrix element and the phonon softening instabilities at the observed CDW modulation vector of q_*CDW*_. The unfolded band structure after the CDW transition in SrPt_2_As_2_ reveals the sizable electron-phonon coupling of the relevant phonon mode. In both cases of SrPt_2_As_2_ and LaPt_2_Si_2_, Pt1 band plays an essential role in the CDW and superconducting transitions, implying that the CDW and the SC coexist in the Pt1 layers.

## Methods

For the total energy band structure calculations, the full-potential linearized augmented plane wave band method implemented in Wien2k package was employed^36^. The generalized-gradient approximation (GGA) was used for the the exchange correlation and the spin-orbit coupling (SOC) was included. For structural optimizations and phonon calculations, the pseudo-potential band method implemented in VASP^37^ and phonopy^38^ were used, respectively. The supercell approach with finite displacements based on the Hellmann-Feynman theorem^39^ was used to obtain the force constants. The pseudo-potential band method implemented in Quantum Espresso was also used to determine the electron-phonon (e-ph) coupling constant *λ*_*p*_ and superconducting parameters^40^. The band-unfolding technique was employed to compare the band structures before and after the CDW modulation^41^,^42^.

## Additional Information

**How to cite this article**: Kim, S. *et al*. The mechanism of charge density wave in Pt-based layered superconductors: SrPt_2_As_2_ and LaPt_2_Si_2_. *Sci. Rep*. **5**, 15052; doi: 10.1038/srep15052 (2015).

## Supplementary Material

Supplementary Information

## Figures and Tables

**Table 1:** Superconducting parameters of LaPt_2_Si_2_.

*N*(*E*_*F*_) (states/eV/f.u.)	*ω*_*log*_ (*K*)	Θ_*D*_ (K)	*λ*_*p*_	*T*_*c*_ (K) μ^*^ = 0.1, 0.13
2.3	117.34	141.78	0.73	4.5, 3.5

*N*(*E*_*F*_), *ω*_*log*_, and Θ_*D*_ are the DOS at *E*_*F*_, the logarithmic average phonon frequency, and the Debye temperature, respectively*. T*_*c*_’s are obtained for two effective Coulomb repulsion parameters μ^*^ = 0.1 and 0.13.
