# Supplementary material for: The mechanism of charge density wave in Pt-based layered superconductors: SrPt2As2 and LaPt2Si2
Source: Sci Rep. 2015 Oct 9;5:15052. doi: 10.1038/srep15052 (PMC4598799; doi:10.1038/srep15052)
Supplement: Supplementary Information [file srep15052-s1.pdf]

Supplemental Material: The mechanism of charge density wave in Pt-based layered superconductors : $SrPt_2As_2$ and $LaPt_2Si_2$

Sooran Kim[1], Kyoo Kim[1,2], and B. I. Min[1]

[1]Department of Physics, Pohang University of Science and Technology, Pohang, 790-784, Korea
[2]c_CCMR, Pohang University of Science and Technology, Pohang 790-784, Korea

1 Structural parameters

Table S1. Structural parameters of $SrPt_2As_2$ without split distortions of Pt1 and As2 atoms (space group *Pmmn*).

a=4.482 Å, b= 4.525 Å, c=9.869 Å			
site	*x*	*y*	*z*
Sr	0.75000	0.25000	0.24690
Pt1	0.75000	0.75000	0.49890
Pt2	0.75000	0.25000	0.88170
As1	0.75000	0.75000	0.99970
As2	0.75000	0.25000	0.62630

Table S2. Structural parameters of $SrPt_2As_2$ with split distortions of Pt1 and As2 atoms (space group *P*-1).

a=4.482 Å, b= 4.525 Å, c=9.869, α=β=γ=90°			
site	*x*	*y*	*z*
Sr	0.75000	0.253367	0.248931
Pt1	0.75000	0.795670	0.497525
Pt2	0.75000	0.247472	0.879795
As1	0.75000	0.749726	0.998933
As2	0.75000	0.283194	0.627811

Table S1 and Table S2 provide lattice parameters of $SrPt_2As_2$, which were used in the calculations. The atomic positions are shifted by (0.5, 0, 0.5) from those of Imre *et al.*.[1] The lattice parameters of $LaPt_2Si_2$ before and after the relaxation are provided in Table S3.

2 Effect of the spin-orbit coupling on the electronic structure of CDW distorted $LaPt_2Si_2$

We have checked the spin-orbit coupling (SOC) effect on the total density of states (DOS) of $LaPt_2Si_2$ after the CDW transition, which is denoted by 3X-LPS in the main text. The total DOSs obtained with the SOC and without the SOC do not have significant difference, as shown in Fig. S1. Therefore, we carried out the electron-phonon coupling constant calculation without the SOC.

Table S3. Structural parameters of $LaPt_2Si_2$. (Space group *P*4/*nmm*) The lattice constants in parentheses are the initial lattice constants from the experiment.[2] The atomic positions in parentheses are the initial atomic positions from idealized tetragonal $SrPt_2As_2$ before the relaxation.[3]

a=4.31559 (4.277) Å, c=9.87452 (9.798) Å			
site	*x*	*y*	*z*
La	0.25000	0.25000	0.743933 (0.74690)
Pt1	0.25000	0.75000	0.000000 (0.00000)
Pt2	0.25000	0.25000	0.379697 (0.38170)
Si1	0.25000	0.75000	0.500000 (0.50000)
Si2	0.25000	0.25000	0.130249 (0.12630)

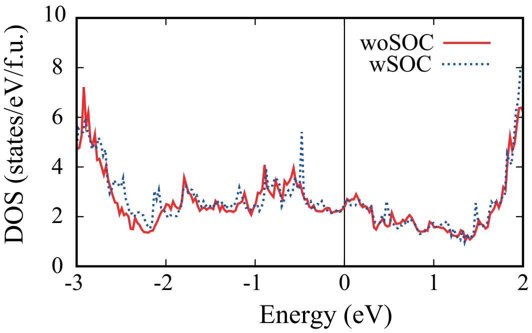

Figure S1. The total DOS of CDW distorted 3X-LPS. The red and blue dotted lines represent the total DOSs with the SOC (wSOC) and without the SOC (woSOC), respectively.

References

1. Imre, A. *et al.* Inkommensurabel modulierte Kristallstrukturen und Phasenumwandlungen - Die Verbindungen $SrPt_2As_2$ und $EuPt_2As_2$. *Z. Anorg. Allg. Chem.* **633**, 2037–2045 (2007).
2. Shelton, R. N., Braun, H. F., and Musick, E. Superconductivity and relative phase stability in 1:2:2 ternary transition metal silicides and germanides. *Solid State Commun.* **52**, 797-799 (1984).
3. Nekrasov, I. A. & Sadovskii, M. V. Electronic structure of novel multiple-band superconductor $SrPt_2As_2$. *JETP Lett.* **92**, 751–755 (2011).
